# COVID-19: an opportunity of systematic integration for Chagas disease. Example of a community-based approach within the Bolivian population in Barcelona

**DOI:** 10.1186/s12879-022-07305-6

**Published:** 2022-03-28

**Authors:** Jordi Gómez i Prat, Hakima Ouaarab Essadek, Juliana Esperalba, Francesc Zarzuela Serrat, Isabel Claveria Guiu, Lidia Goterris, Ricardo Zules-Oña, Estefa Choque, Conxita Pastoret, Natàlia Casamitjana Ponces, Juan José de los Santos, Jordi Serrano Pons, Aurore Dehousse, Pedro Albajar-Viñas, Tomàs Pumarola, Magda Campins, Elena Sulleiro

**Affiliations:** 1 Public Health and Community Team (eSPiC), Unit of Tropical Medicine and International Health Drassanes-Vall d’Hebron (UTMIHD-VH), PROSICS, Servei de Medicina Preventiva, Carrer de Sant Oleguer, 17, 08001 Barcelona, Spain; 2 Microbiology Department, Vall d’Hebron University Hospital, Barcelona, Spain; 3 Preventive Medicine and Epidemiology Department, Vall d’Hebron University Hospital, Barcelona, Spain; 4 Asociación de Amigos de las Personas con la Enfermedad de Chagas (ASAPECHA), Barcelona, Spain; 5 Departament de Salut, Generalitat de Catalunya, Banc de Sang i Teixits de Catalunya, Barcelona, Spain; 6 Fundación Mundo Sano-España, Madrid, Spain; 7 Universal Doctor, Barcelona, Spain; 8 Department of Control of Neglected Tropical Diseases, World Health Organization, Geneve, Switzerland

**Keywords:** Chagas disease, COVID-19, Community-based approach, Opportunity of systematic integration, Bolivia, Barcelona

## Abstract

**Background:**

As a Neglected Tropical Disease associated with Latin America, Chagas Disease (CD) is little known in non-endemic territories of the Americas, Europe and Western Pacific, making its control challenging, with limited detection rates, healthcare access and consequent epidemiological silence. This is reinforced by its biomedical characteristics—it is usually asymptomatic—and the fact that it mostly affects people with low social and financial resources. Because CD is mainly a chronic infection, which principally causes a cardiomyopathy and can also cause a prothrombotic status, it increases the risk of contracting severe COVID-19.

**Methods:**

In order to get an accurate picture of CD and COVID-19 overlapping and co-infection, this operational research draws on community-based experience and participative-action-research components. It was conducted during the Bolivian elections in Barcelona on a representative sample of that community.

**Results:**

The results show that 55% of the people interviewed had already undergone a previous *T. cruzi* infection screening—among which 81% were diagnosed in Catalonia and 19% in Bolivia. The prevalence of *T. cruzi* infection was 18.3% (with 3.3% of discordant results), the SARS-CoV-2 22.3% and the coinfection rate, 6%. The benefits of an integrated approach for COVID-19 and CD were shown, since it only took an average of 25% of additional time per patient and undoubtedly empowered the patients about the co-infection, its detection and care. Finally, the rapid diagnostic test used for COVID-19 showed a sensitivity of 89.5%.

**Conclusions:**

This research addresses CD and its co-infection, through an innovative way, an opportunity of systematic integration, during the COVID-19 pandemic.

## Background

The COVID-19 pandemic has represented a persistent sanitary challenge since its inception, also for patients with parasitic diseases. This is notably the case of Chagas disease (CD) coupled with the acute COVID-19, with myocarditis [1] and other clinical manifestations [2] as well as the post-acute COVID-19 syndrome [3–6].

CD is mainly a chronic infection that principally causes cardiomyopathy and can also cause a prothrombotic status, putting the population infected with the parasite that causes it, *Trypanosoma cruzi,* at risk of presenting severe COVID-19 [2, 7]. One of the main challenges of CD control is the detection of cases. Worldwide it is estimated that < 10% of the estimated 6–7 million infected people have been diagnosed, leading to a high epidemiological silence [8–10]. CD, originally confined to the Americas, has been detected in up to 42 countries, due to the increased population movements of the last decades [11]. Since the first case was detected in Europe, 42 years ago [12] up to seventeen European countries have diagnosed CD cases, with the highest prevalence in Spain [13]. By contrast, in less than a year, the SARS-CoV-2 infection has rapidly spread in all world countries facilitating the epidemiological overlapping, co-infection and co-morbidiy.

CD is one of the Neglected Tropical Diseases (NTDs), as labeled by the World Health Organization (WHO) in 2005 [14]. Despite the different public health initiatives of the last years at national and international level [15] and the existence of several CD patients organizations, gathered in an international federation (www.findechagas.org), affected people still struggle to get the attention they need, financial resources to mobilize, network and make their case in the micro politics climate in which health care is brokered. The “social determinants of health” are defined by WHO as “the conditions in which people are born, grow, live, work and age, and the wider set of forces and systems shaping the conditions of daily life”. They have had a great impact on CD patients since in most cases, it is more likely to affect disadvantaged people who are economically and politically vulnerable [16, 17]. This is especially the case of migrant populations facing multiple challenges in their access to healthcare services, including diagnosis, treatment and follow-up. Besides, psycho-emotional and socio-anthropological barriers are extremely significant in the case of CD [18–22], and specifically the fear of the disease and the social stigma it might trigger [23].

Other barriers, related to administrative processes and healthcare access, are also quite important since the majority of the potentially affected population is immigrant and most of the time, not familiar with the administrative procedures of their host countries [24]. This calls for a multidimensional analysis and approach [25], that the public health & community team (eSPiC) of the Unit of Tropical Medicine and International Health Drassanes-Vall d’Hebron (UTMIHD-VH) has started to put in place since 2008. It has indeed been working with the Latin American community in Barcelona to promote and enhance CD diagnosis [26]. Since 2014, the eSPiC/UTMIHD-VH has implemented community screenings of *T. cruzi* infection for people of Latin American origin living in Catalonia. In parallel, it also entered into a partnership with the General Consulate of Bolivia in Barcelona (Table 1) in order to promote community-based actions based on information, education and communication (IEC) components, reducing underdiagnosis within the Bolivian community (historically the community with the highest rate of *T. cruzi* infection), provide quality care, follow up to the infected individuals and, eventually, prevent congenital transmission [1, 2, 25–31].

**Table 1:** Key methodological components of the study background led by UTMIHD-VH (2008–2019) in the city of Barcelona

Key components of the study	Design, development and implementation	Years and periods of activity
*Study background*		
Human resource mobilization, community participation and network of partners and stakeholders	Support to the Latin American migrant population and creation of associations of affected population: Support to the creation of the Association of friends of people with Chagas disease—ASAPECHA—in Barcelona Officially registration of ASAPECHA Progressive set up of a group of stakeholders and partners to ensure communication, liaison and identification of key health actions for Latin American migrant population Joint decision makings and implementation with all involved actors [26]	(2007–2008) (2008) Continuous implementation (2008–2020)
Health Education	Catalonian Expert Patient Programme for Chagas Disease Developed in collaboration with members of ASAPECHA, Elaboration of files, PowerPoint presentations and other communication materials, design sessions [42] BeatChagas platform [23]	Implementation sessions (2011–2020) Design and development of IEC materials and platform (2011–2021)
Mobilization of the population at risk of CD	Contact and partnership with the General Consulate of Bolivia in Barcelona: Configuration of a work team with key specific actors and joint decision making^a^	Work meetings, events participation and community interventions (2014–2020)
Human mobilization of Latin American general population and strategic interventions	Contact and partnership with Latin American sport and culture associations in Barcelona Participation in cultural events and IEC interventions [26] In situ interventions, with IEC and blood screening [27, 28]	Participation 2012–2020 2014–2020

^a^List of actors: General Consulate of Bolivia in Barcelona, ASAPECHA, Unit of Tropical Medicine and International Health Drassanes-Vall d’Hebron (UTMIHD-VH), Blood and Tissue Bank of Catalonia (BTBC)

In light of the pandemic, the idea of a community-based approach of CD and COVID-19 arised as an opportunity of systematic integration (OSI). The “community-based” term refers to a philosophical approach in which communities play an active role, highlighting and addressing the issues they identify as important to them. Communities are notably encouraged to actively design, develop and deliver their own prevention and intervention strategies, which challenges community members to identify what the issues are and work together to address those issues [32]. OSI are strategies developed in order to face the challenges of coinfection and co-morbidity between diseases. Worldwide, they have progressively been implemented since 2007 and proved to have positive outcomes on different diseases. Among OSI examples we notably find the following: diagnosis of haemoparasites infection (*Plasmodium* spp., filariasis spp. and *T. cruzi*) through malaria films [33, 34]; screening of the Human Immunodeficiency Virus (HIV), Hepatitis B (HBV), Syphilis and CD at birth [35]; screening of opportunistic infections that define AIDS condition [36, 37]; dual epidemiology approach (with communicable and noncommunicable diseases) in the screening of chronic diseases that can cause chronic cardiomyopathy (CD, rheumatic heart disease, hypertension, diabetes, cardiorenal syndrome…) [8, 38].

OSI used by the CD Control Programme of WHO could apply to the complex situation generated by the COVID-19 syndemic [39] and co-infection with *T. cruzi* infection. The opportunities are given in the coincidence in space and time of two or more existing processes or elements. The opportunities already exist, and the challenge is to identify them. The OSI can offer the possibility to increase detection, diagnosis confirmation, care, prevention, control and cost-effectiveness. Once an OSI has been identified, the non-integration can be considered as a missing opportunity or even a bad practice [8].

We drew on previous community-based experiences, including participative-action-research components already known and well accepted by health professionals and community leaders and members [40].

The four specific objectives of the study were:

1. To assess the percentage of people with Bolivian nationality previously screened for *T. cruzi* infection in Barcelona and determine where the diagnosis of *T. cruzi* infection took place.
2. To diagnose *T. cruzi* infection cases, determine the prevalence of SARS-CoV-2 infection and co-infection among Bolivian nationals in Barcelona.
3. To assess the feasibility of an OSI based on a community-based approach for SARS-CoV-2 and *T. cruzi* infections and co-infection.
4. To determine the performance of a serologic rapid diagnostic test for COVID-19 used in the integrated approach.

## Methods

**Fig. 1:**
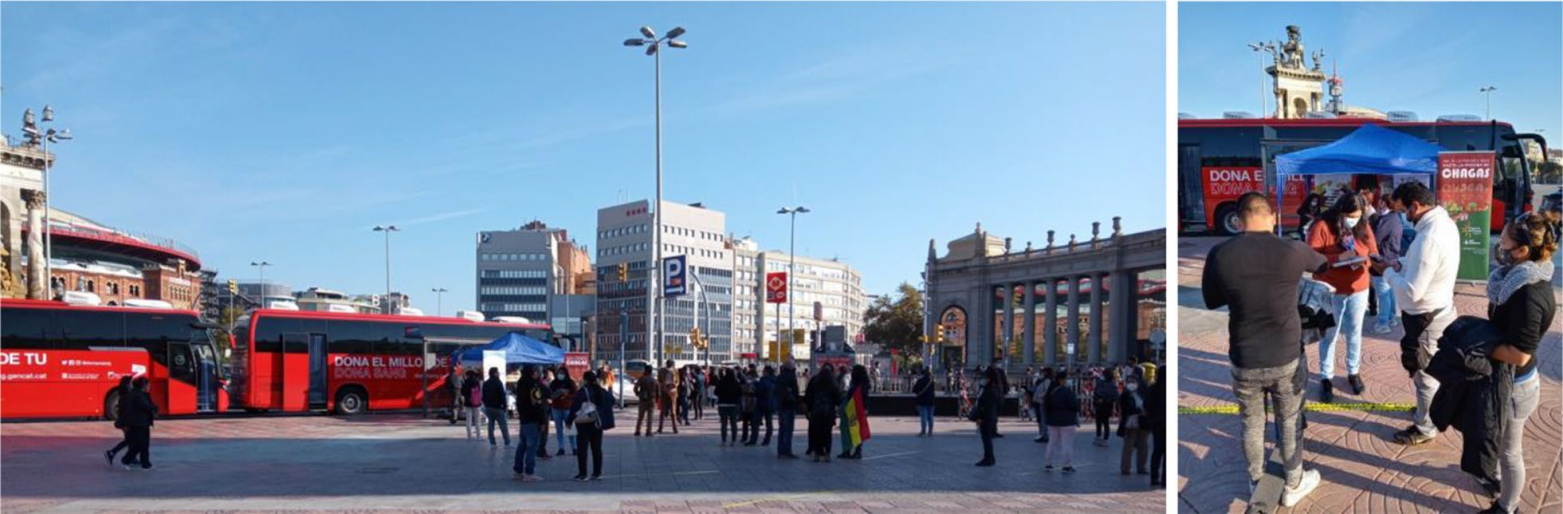
Left: General view of the field study place, with two mobile units and a stand. Right: Community health workers and members of the association of people affected by Chagas disease (ASAPECHA) offering information and infection screening to people that came to vote. Photo: Jordi Gómez i Prat, 2020

The work was carried out in the Metropolitan area of the city of Barcelona, Autonomous community of Catalonia, Spain, among the Bolivian community during the national Bolivian elections organized on 18th October 2020 (Fig. 1). In 2019, Catalonia had a population of 7,619,494 inhabitants, with 1,159,427 foreigners, 29,570 Bolivian nationals of which 8495 lived in Barcelona city [41].

Key background methodological components this study is based on are specified in the Table 1. Sequentially, the UTMIHD-VH decided to use the 2020 Bolivian national elections for the first time to target the community. This was enabled by the long lasting collaboration and work of awareness raising with official representatives of the Consulate of Bolivia. In the context of COVID-19 restrictions, the Bolivian elections were the only event that could attract the highest number of Bolivians in the metropolitan area of Barcelona. All Bolivian nationals were eligible to participate in the screening.

Participants were recruited by community health agents and peer-to-peer educators from ASAPECHA Barcelona (association of friends of people with Chagas disease of Barcelona) who had been previously trained on the multidimensional approach of the disease. Their main focus was on the psycho-social aspects that make access to CD diagnosis difficult. They interviewed the participants and informed them about CD, the barriers to healthcare access, and the importance of CD diagnosis and treatment. The interviewers also gave them information specifically relevant to their own case/history.

Additionally, participants were asked if they had already heard of CD before, and if they had already been tested for it and where. Afterwards, the participants who had never been tested for CD before were offered the chance to get screened in situ for both the *T. cruzi* and the SARS-CoV-2 infection, taking advantage of a unique blood extraction, taken by the mobile teams of the Blood and Tissue Bank of Catalonia (BTBC). Key methodological components of the community-based approach intervention are summarized in Table 2.

**Table 2:** Key methodological components of the community-based approach intervention of CD and COVID-19 in the city of Barcelona (2020)

Key components of the study	Design, development and implementation (description of methodological elements)	Periods of activity
2020 Community Health Action/intervention/strategy		
Mobilization of human resources, partners and stakeholders	Coordination with the General Consulate of Bolivia in Barcelona and the Plurinational Electoral Body of Bolivia^a^: Agreement for the implementation of the CD and COVID-19 screening intervention in the framework of the national elections of Bolivia	August–September 2020
Human resource mobilization	Coordination with the Blood and Tissue Bank of Catalonia Discussion and design of the intervention protocols (in the context of the COVID-19 pandemic)	August–September 2020
Human resource mobilization and health education	Coordination with ASAPECHA Agreement to carry out the study; identification of human resources needs and training on COVID-19	August–September 2020
Human resource mobilization, IEC and screening intervention	Implementation of the field strategy of screening The resources included mobile units, public health experts, nurses, community health agents and peer educators	18 October 2020
Laboratory diagnosis and healthcare	Processing of laboratory results and contact of infected population Medical care and follow-up of infected population	October–November 2020 Since November 2020

^a^The Plurinational Electoral Body is an electoral body of the Plurinational State of Bolivia. It is made up of the Supreme Electoral Tribunal, the Departmental Electoral Courts, the Electoral Courts, the elected Juries and the Electoral Notaries

The informative and educational activity was successfully carried out in the waiting line before entering to vote, by peer educators. After voting, they welcomed the volunteers to participate in the study. Sociodemographic data gathering from participants was done by community health agents before blood extraction. The data gathered, as well as any referred symptomatology, was verified and registered in the SAP NetWeaver. This computer software was previously reconfigured so that the two screening tests could be requested in a single request, unequivocally for each person.

The serologic screening of the *T. cruzi* infection was done with a chemiluminescence technique using recombinant antigen (Elecsys Chagas. Roche). Following the WHO diagnostic recommendations for the diagnosis of chronic CD, an ELISA technique using a native antigen (Ortho *T. cruzi* ELISA, Ortho diagnostics) was also performed in positive samples. Patients with both positive serological results were considered infected. If both tests were negative, infection was ruled out. Sera with a positive recombinant ag test and a negative ag test were considered discordant. To test SARS-CoV-2 infection, the sera underwent a determination of total antibodies by chemiluminescence (Elecsys Anti SARS-CoV-2. Roche). When positive, a detection of specific anti-IgG was confirmed by chemiluminescence (LIAISON^®^ SARS-CoV-2 S1/S2 IgG. Diasorin).

Additionally, sera with positive total antibodies were tested with a rapid immunochromatographic assay (Fastep COVID-19 IgG/IgM Rapid Test Kit. Grifols), which enables detecting separately both IgG and IgM.

People with a positive result on one of the two screening tests were contacted by phone and referenced to the UTMIHD-VH to initiate bio-psychosocial care, including antiparasitic treatment, follow-up and determine if the cardiac, digestive and/or neurological systems were affected and to what extent. In case of a positive serological result for COVID-19, patients with *T. cruzi* infection were informed about possible future health implications and their right to be vaccinated in priority for COVID-19. The patient's condition was registered in the electronic medical records (software SAP) of the Catalan Health Institute and in the App called “La meva Salut” of the Catalan Health Service, Department of Health of the Government of Catalonia (DH/GC) (https://lamevasalut.gencat.cat/es).

All the subjects who were interviewed gave verbal consent to participate in the in situ screening. This is part of the conventional protocol of our clinics to screen susceptible CD individuals and provide healthcare to diagnosed patients. The procedures performed during the screening are the ones recommended by WHO. No data containing personal or identifying information from the participants has been published.

The resources used, including infrastructure, instruments, consumables, human resources and time, were calculated based on the following: (i) experience of previous community screening actions with the 29,570 Bolivian residents in Catalonia; (ii) specific COVID-19 prevention measures implemented by DH/GC; (iii) voting time period (from 8 to 17 h); (iv) availability of existing resources on the day of election (Sunday 18 October 2020).

The statistical analyses were performed using Stata v14 (StataCorp. 2015. Stata Statistical Software: Release 14. College Station, TX, USA: StataCorp LP). Median and interquartile range (IQR) as well as proportions were used for the description of quantitative variables and qualitative variables, respectively. To assess the association between the different variables, the Chi squared test and the Fisher’s exact test were performed.

## Results

### Percentage of people with Bolivian nationality previously screened for *T. cruzi* infection in Barcelona and determination of the place of infection diagnosis

Out of the 7421 people censused in the Plurinational Electoral Body of Bolivia and having the city of Barcelona as a voting place, 4866 (65.57%) participated in the Bolivian national elections and 1200 (16.17%) were finally informed and interviewed at random at their arrival at the polling place. In order to know the percentage of people screened for *T. cruzi* infection and place of diagnosis, 604 people, out of the 1200 people informed and interviewed, were specifically asked if they had undergone a previous screening and 272 (45%) said no. The 332 people (55%) who answered positively were also asked whether and where they were diagnosed and 269 (81%) answered that it was in Catalonia and the rest (19%) in different Bolivian departments. All samples were aleatory and statistically representative, with a confidence interval of 95% and a precision/accuracy of ± 5% units.

### Prevalence of *T. cruzi* and SARS-CoV-2 infections and co-infection rates in the migrant population with Bolivian nationality in Barcelona

A total of 299 people were finally screened for *T. cruzi* and SARS-CoV-2 infection. The median age was 43 years (IQR 33–53). Among those screened, 65.9% (197/299) were women and all participants were born in Bolivia (Table 3).

**Table 3:** Demographic characteristics of the Bolivian population that underwent in situ *T. cruzi* and SARS-CoV-2 infections screening

	n	%
Gender		
Female	197	65.9
Male	102	34.1
Age (years)		
< 30	23	7.69
30–39	85	28.43
40–49	107	35.79
50–59	64	21.40
> 60	20	6.69
Country of birth Bolivia	299	100
Total of screenings	299	100

Out of the 299 screened people for *T. cruzi* infection, 55 (18.3%) of them presented a positive result of the chemiluminescence and ELISA tests, 3.3% [10] presented discordant results (a positive chemiluminescence result followed by a negative ELISA result) and 78.3% (234) presented both negative results. A total of 61.8% (34/55) of the positive results were in women, but 17.3% (34/197) of all screened women presented positive results, while 20.6% (21/102) of all screened men presented positive results.

The median age of people with positive serologic results was 48.4 years (IQR 39–57.8). Among all people with positive results, the age decade with the highest prevalence of infection was 40–49 years old (36.4%) (Table 4).

**Table 4:** Description of the demographic characteristics of the Bolivian individuals that tested positive to the screening tests for *T. cruzi* and SARS-CoV-2 infections performed during the community interventions

	*T. cruzi* positive results		SARS-CoV-2 positive results	
	n	%	n	%
Positive	55	18.3	67	22.3
Female	34	61.8	41	61.2
Male	21	38.2	26	38.8
Age (IQR)	48.4 ± 9.4		42.5 ± 9.6	
Age (years)				
< 30	0	0.0	7	10.5
30–39	10	18.2	13	19.4
40–49	20	36.4	33	49.2
50–59	17	30.9	11	16.4
> 60	8	14.5	3	4.5

**Fig. 2:**
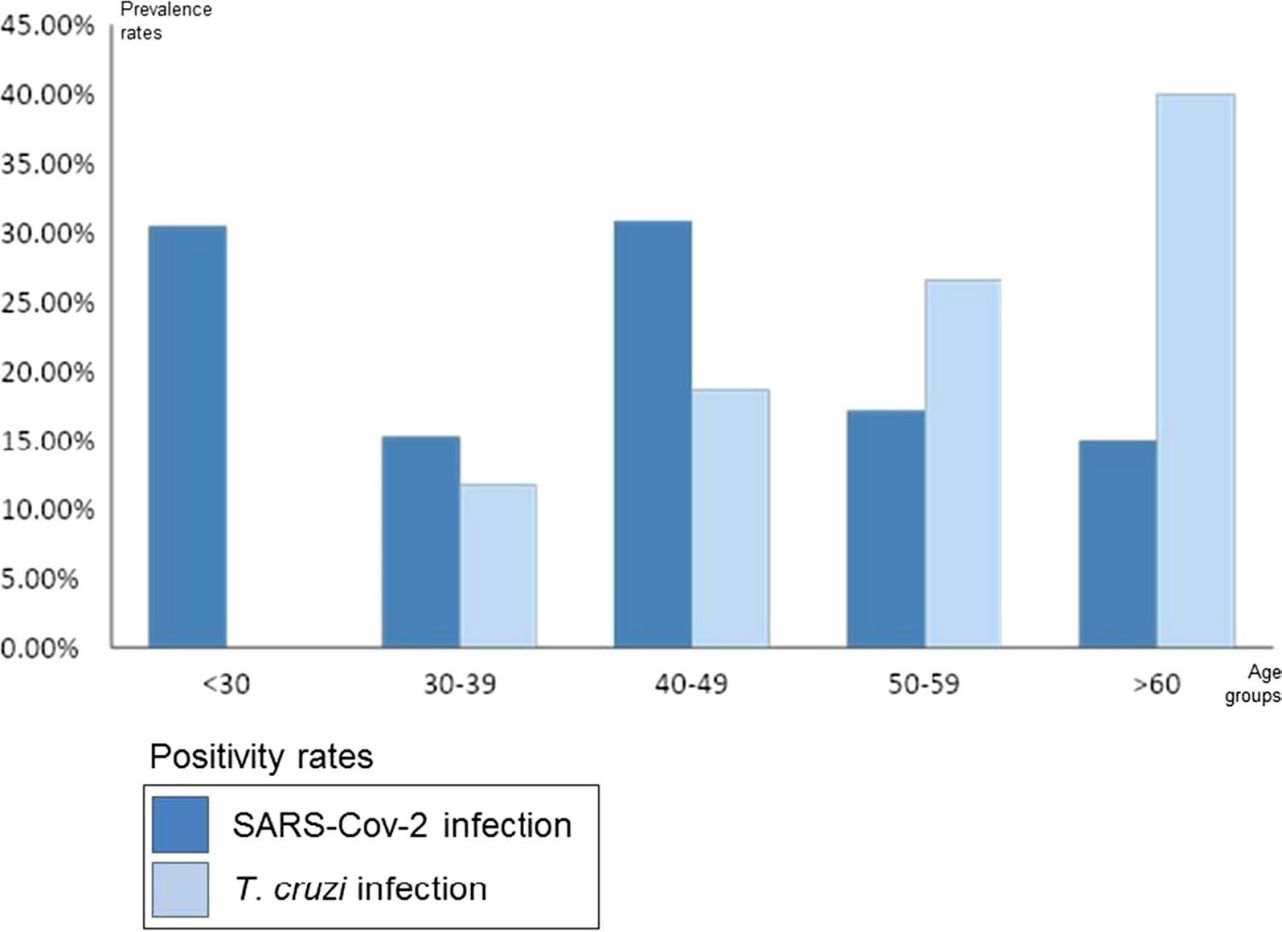
*T. cruzi* and SARS-Cov-2 infection rates by group age

In relation to the decade of age, we found a total of 40.0% (8/20) of positive results in those older than 60 years, 26.6% (17/64) in those between 50 and 59 years, 18.7% (20/107) in those between 40 and 49 years, 11.8% (10/85) in those between 30 and 39 years and no cases were found (0/23) among those < 30 years of age (Fig. 2). The difference between decades was statistically significant (Chi: 0.002/Fisher: 0.001).

The determination of SARS-CoV-2 IgG was positive in 22.3% (67/299) of the total population. A total of 61.2% (41/67) of the positive results were in women; 20.8% (41/197) of all screened women presented positive results, while 25.5% (26/102) of all screened men presented positive results.

The age group with the highest number of positive results of the screening test was 40–49 years (49.2% of the total positive results) (Table 4). The study found a positivity rate of: 15% (3/20) of people > 60 years old; 17.2% (11/64) of people between 50 and 59 years old; 30.8% (33/107) for those between 40 and 49 years old; 15.3% (13/85) among the people between 30 and 39 years old and 30.4% (7/23) among those < 30 (Fig. 2). There is hence a great difference between age categories (Chi: 0005).

Finally, it is interesting to highlight that out of the 299 screened people 18 individuals that tested positive for *T. cruzi* infection also tested positive for anti-SARS-CoV-2, which represents a coinfection prevalence of 6%.

### Feasibility of an OSI based on a community-based approach for SARS-CoV-2 and *T. cruzi* infections and co-infection

After voting, 299 participants who had not been previously tested accepted to be screened in situ for both the *T. cruzi* infection and the SARS-CoV-2 infection. This represents an acceptability rate of 55% (299/540), since 45% (or 540 out of the 1200) of the people that were interviewed had never been previously tested and considered it to be a good opportunity.

Based on the specified methodological calculations, the following resources were finally used: two mobile units of the BTBC, with two usable stretchers per unit; one physician and one nurse, public health experts, coordinating the whole implementation; four nurses for the blood extraction distributed in the two mobile units and one tent; five community health agents from UTMIHD-VH and seven peer educators, (members of the ASAPECHA Barcelona) doing IEC and recruitment.

Table 5 shows that an integrated approach of both SARS-Cov-2 and CD per patient takes on average 20 min, in comparison to 15 for a first consultation for CD, which represents an additional 25% of time of doing a unique CD approach.

**Table 5:** Time per patient needed for the integrated approach for CD and COVID-19

	Time dedicated to Chagas disease (in minutes)	Time dedicated to COVID-19 (in minutes)	Total time spent per person (in minutes)	Percentage of time increase
Peer educators	1–5	No additional time needed	1–5	0
Community health agents	3–5	2–5	5–10	40–50
Blood extraction	5	No additional time needed	5	0
Total	9–15	2–5	11–20	25

The study also showed that the use of the SAP NetWeawer program eased the process. The laboratory work was quite speedy since it consisted of tagging and processing the samples at the same time. Those tasks were carried out by a team as well as an automated chemiluminescence immunoassay, which enables suctioning the collected material directly from the extraction tube. It is hence time saving since there is no need to aliquot or centrifuge the samples.

### Performance of a rapid diagnostic test for COVID-19 used in the integrated approach

A rapid diagnostic test (RDT) was carried out on the 68 sera that presented positive antibodies to COVID-19 in the chemiluminescence immunoassay (CHIA). The following results were obtained: 89.5% (60/67) tested positive for IgG and 61.2% (41/67) tested positive for IgM. This showed a high performance of the rapid tests. Due to the high sensitivity of the CHIA, none was carried out on the negative sera.

## Discussion

**Fig. 3:**
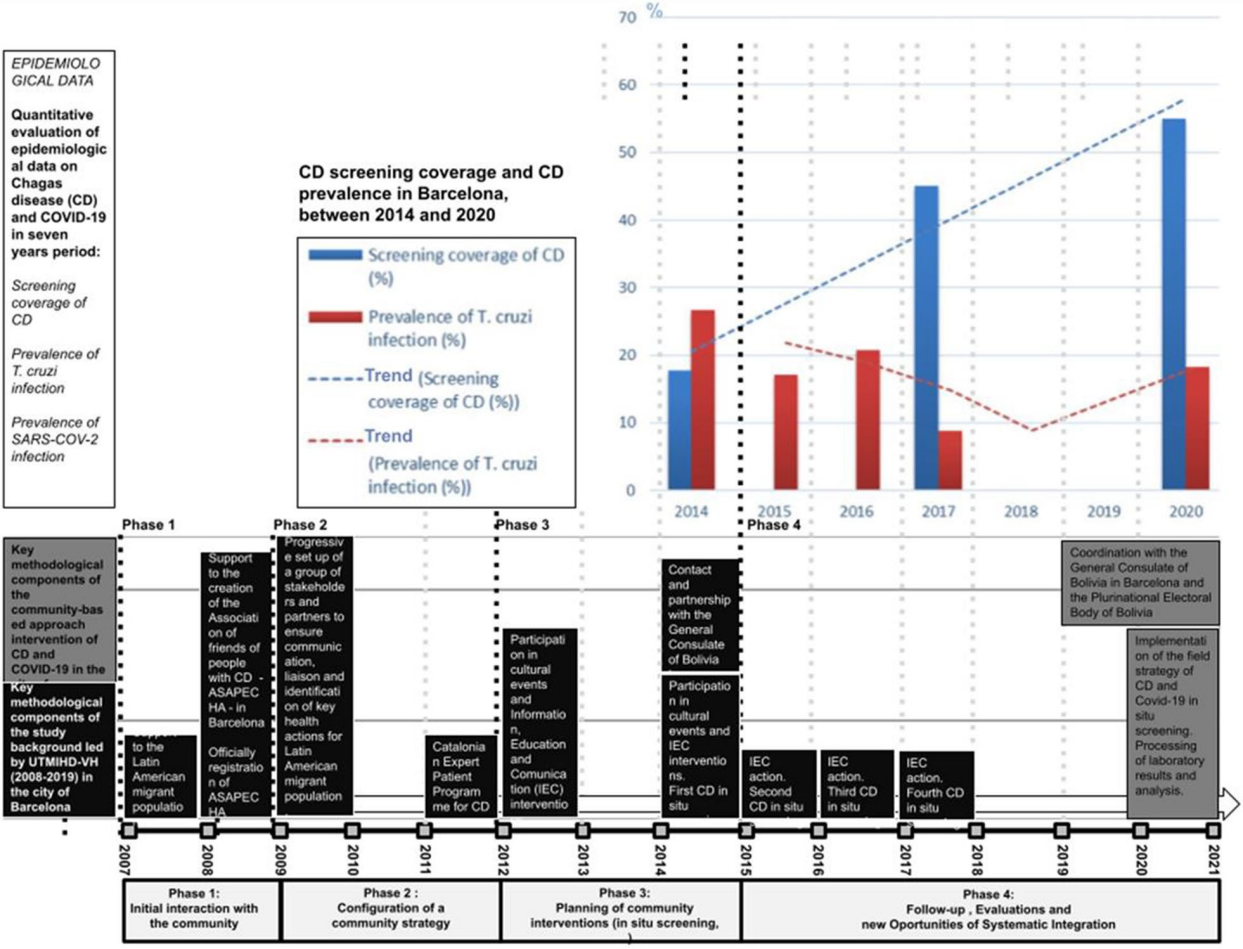
Timeline of project interventions in the city of Barcelona between 2007 and 2020, with epidemiological Chagas disease data between 2014 and 2020

This article describes an innovative and pioneer integrative approach for the field of healthcare. Through a community approach, two diseases—COVID 19 and CD—were tested on one single occasion, enabling potential infected people to access healthcare for the first time. This work was based on fourteen years of previous work (Fig. 3), during which awareness raising activities and discussions took place with a wide array of actors going from immigrants, health and civil society organizations to governmental institutions based in the city of Barcelona. The collaboration of more than five years with the Bolivian Consulate in Barcelona was one of the key elements to be able to carry out this study and work with the population at higher risk.

Previous in situ screening actions were performed between 2014 and 2017 at the occasion of different Bolivian community events and previously described by our group [27, 28]. The technical appoach of community actions on the recruitment of patients for screening tests has also been described at the occasion of: workshops performed in 2012 and in 2013 [43] in Bergamo, Italy,); informative group talks in Madrid, Jerez de la Frontera and Alicante, Spain, between 2007 and 2010 [44]; and the combined use of informative brochures and workshops at Bolivian community events in the case of Munich, Germany, in 2013 and 2014 [45]. Those previous experiences taught us that having people specifically trained on CD and its different components before undertaking a study was key to its implementation and development. It enables a “conscious action”, in which the actors are fully aware of all the socio-psychological dimensions of CD and know how to help and lead people to overcome them [46]. Besides, the fact that the people raising awareness and playing the part of reaching to the people at the voting site were from the organized civil society (as ASAPECHA members) with a trained background (as Expert Patient Programme for CD), created an in-depth feeling of equality and trust for the people. That was determinant for the good implementation of the study [42].

The implementation of an OSI approach highlights several benefits [8]. It optimizes time, human capital, economic resources, IEC materials, infrastructure, as well as instruments and consumables, especially in the laboratory work. It increases efficiency and effectiveness, and from the public health point of view, the failure to identify and take advantage of existing OSI can be considered a major missing opportunity.

This operational research represents a real turning point for our team in Barcelona and more generally, the field of CD. The public health field is characterized by a search for “impact and effectiveness of programs”, a space in which rational choice calculation operates as to determine where to allocate resources and where the benefits of an envisaged intervention would be highest compared to what would happen if such intervention did not take place [47]. From a public health policy perspective there is hence no incentive and no interest in mobilizing extensively for a NTD such as CD.

Out of the 604 people asked about previous *T. cruzi* infection screening, 332 (55%) of them answered affirmatively. Since the study was eventually able to screen 299 people, it reached one of the highest acceptability rates (55%) for this type of study as well as the largest *T. cruzi* infection screening coverage in the world right now [10]. Considering that in 2014 the coverage rate was 17.7% [27] (Fig. 3), significant progress was made, as also witnessed in our study of 2017, where the coverage rate was 45.4% [28]. Another key finding is that 81% of the people with Bolivian nationality have been diagnosed in Catalonia. Indeed, since 2005, active and passive screening have been progressively implemented in the territory, with 33% of diagnoses done in CD-specialized health facilities; 21.2% community health actions; 8% at primary health level and 37.8% in a miscellaneous of additional places [28]. The activities implementation of the blood and tissues bank of Catalonia since 2005 and the programme of congenital CD since 2010 have played a significant role.

The prevalence of CD among the people that were tested was 18.3%, a higher value than the 8.9% we found in 2017 in Barcelona (Fig. 3). Other studies also showed similar prevalences, between 9.3% and 27.7% [43–45, 48]. A similar random effect pooled prevalence of 18.1%, within the Bolivian population, and lower prevalence of 4.2% within Latin American population, were both calculated in 2015, from 18 studies of five European countries, [49]. It marked the first time a population sample was obtained in a national election with mandatory voting. So, with most probable values of infection closer to reality. On the other hand, the Bolivian population shows once more one of the highest *T. cruzi* prevalence rates of infection in the world.

The detection of IgG antibodies indicates exposure to the SARS-CoV-2 virus and permits to estimate the prevalence in a specific population. Until now, no prevalence studies have been done on SARS-CoV-2 and migrant communities. The prevalence of 22.3% of the virus among the Bolivian population is way higher than the one found in the overall population of the province of Barcelona (6.8%) or the one found in Spain’s foreign population (5.7%) [50]. That can notably be explained, as stated earlier, by sociocultural factors such as work and living conditions in which lationamerican immigrants often find themselves in their host country. Some interviews done during the study also highlight that members of that community are more likely to gather—fueling the spread of the virus—and be fearless of the disease [51]. Because of their economic status, potentially infected people tend to live in dwellings and work in positions with major exposure to the COVID-19 virus. They are part of a category of people that has a limited capacity to take preventive measures, leading to a higher prevalence of SARS-CoV-2 infection among them [52]. The 2003 outbreak of SARS also taught us that the attitude when confronting an infectious disease depends on the understanding and the level of panic within the population itself. They are hence important variables when trying to prevent the spread of the virus [53, 54].

Finally, it is interesting to look at the age variable when studying the CD prevalence. As we saw, the range with the highest number of cases was the one of people 40–49 years old. This is significantly different from our last study [26], where the highest number of affected individuals were 50–59 years old. However, when looking at the prevalence rate per age range we found that the highest value was among the group of individuals over 60 years old, followed by the 50–59 years old, and so on. This reflects the fact that over the last decades, there has been an improvement in the transmission control of CD in Bolivia.

On the other hand, Pollan [50] showed that the age variable does not play a role when it comes to SARS-CoV-2. However, as shown earlier, there is a statistical difference in our study—with significantly more cases among people 40–49 years old and under 30 years old. This fact could be explained by the dynamics of socialization of these groups.

Because this study was based on one single event—national election day in Bolivia—the screening was conducted on a high number of people in one single day: 1200. Out of those, we managed to fully test 299 people. In comparison, in two of our previous studies, in both 2014 and 2017, we respectively tested 169 and 271 people in 2 days [28]. As other elements of comparisons, we can also think of other community studies that took place in the city of Bergamo, Italy, and in the city of Madrid, Spain. In Bergamo, 1,305 people were screened as a result of different workshops carried out over a year (3–4 people per day) [43] and in Madrid, 352 people were tested in 44 workshops carried out over two and a half years (eight people per workshop) [44]. The number of 299 screened people represents an acceptability of 55% (299 out of 540 people), which demonstrates good feasibility and acceptability of community intervention. Because only one fourth of the people who participated in the elections were interviewed (1200/4866), the study shows that four times the number of resources would have been needed to interview all participants.

Community interventions are group activities planned and carried out in a collaborative manner in the community and aimed at improving health and well-being through the empowerment of the affected people [55]. The share of people that agreed to be screened was quite similar to the ones we (and other groups) had in previous studies conducted on people potentially infected with *T. cruzi*. It has been agreed that the psycho-emotional and socio-anthropological factors are highly relevant to access care. This work fundamentally contributes to reconstructing perceptions around CD and even COVID-19 on the part of those affected, the general population and the health professionals themselves.

Another major takeaway of this study is that following this same model of communitary actions, both in rural and urban areas, it is possible to really integrate people in the development and execution of health strategies on the long run. People become truly aware of the risks they incur and of the steps they need to take to take care of their own health [56].

Other key elements to facilitate the recruitment of people and enhance access to diagnosis and care were the creation of a multidisciplinary community health team, involving Primary Health Care, as well as the use of community strategies based on studies about the socio-anthropological and psychological aspects of the disease [28].

As the results show, the investment in human capital as well as in logistics was minimal for a comprehensive but successful approach of promotion, prevention and care. Working with people already knowledgeable about CD enabled us to optimize the time dedicated to each person while giving excellent care and duly informing about the disease and its specifics.

A significant experience of the study was the reprogramming of the lab software (SAP NetWeaver) to process simultaneously the two screening tests of these two different infectious diseases in a single request. The current digital health revolution facilitates the invention of new decision support systems and can be a facilitator instrument to implement different OSI with a systemic approach. The technological evolution in screening, collection of data, diagnosing, treatment, storing, processing as well as the use of deep learning can definitely lead to improved health indicators [57].

Despite the fact that the best sensitivity to detect SARS-CoV-2 antibodies is obtained through conventional serological tests, this study has shown a sensitivity rate of RDT above 89%. This, coupled with the fact that they are easy to use and that results are obtained rapidly, put them as a good option to use in situ of community studies [58].

Despite its devastating consequences, the COVID-19 pandemic gave new opportunities worldwide in a wide array of matters, opening doors for new ways of thinking and acting. This work relates a concrete example of our capacity to bring about inspiring and practical solutions at a local level when facing uncertainty. As such, the study that this paper presents, was enabled by the crisis context itself. COVID-19 appeared as an opportunity window for CD, setting it on the agenda of the Bolivian consulate. John W. Kingdon [59] identifies an opportunity window when three autonomous and different streams meet: problem stream, policy stream and political stream. Applied to our study, this model highlights CD as the problem stream—there is an issue and something can be done to fix it; the people that mobilize in order to address the problem—notably the UTMIHD-VH—as the policy stream; and finally, the Bolivian national elections as the political stream, where ideas, alternatives and solutions to the situation are presented. In the case of the following study, it is clearly COVID-19 that acted as a “window opener” for CD to be tested massively on Bolivian nationals.

## Conclusion

This study shows one path to fight the current underdiagnosis of CD through a community-based approach, encompassing fully the multidimensional understanding of CD. Additionally, it shows that systematic integration is a major tool to fight and manage SARS-CoV-2 infection between community health teams, Primary Health Care level and second and third health level, while at the same time allowing access to diagnosis for other diseases.

In Europe, the city of Barcelona holds one of the highest coverage of *T. cruzi* screening and diagnosis of the migrant Bolivian population. Most of the patients undergo screening and are given a diagnosis at their arrival in the metropolitan area of Barcelona, confirming that the area has a strong and efficient diagnosis and prevention system of CD.

Because of their economic and social characteristics, the prevalence of *T. cruzi*, SARS-CoV-2 infection and their co-infection among Bolivian nationals in Barcelona is much higher than within the local general population.

Detection of a NTD such as CD is one of the biggest challenges of the implementation of the 2021–2030 WHO roadmap [60]. This study illustrates the benefits of this type of action in the fight against SARS-CoV-2 infection by a multidisciplinary team and rapid diagnosis tests. The lessons learned have shown how to replicate an OSI based on a community-based approach on other similar occasions and in other places.

## Data Availability

The datasets used and analysed during the current study are available from the corresponding author on reasonable request.

## Abbreviations

CD: Chagas disease
WHO: World Health Organization
NTD: Neglected tropical diseases
UTMIHD-VH: Unit of Tropical Medicine and International Health Drassanes-Vall d’Hebron
eSPiC: Public Health and Community Team
ASAPECHA: Asociación de Amigos de las personas afectadas por la enfermedad de Chagas—Associations of Friends of Chagas affected patients
BTBC: Blood and Tissue Bank of Catalonia
IEC: Information, education and communication
OSI: Opportunity of systematic integration
IQR: Interquartile range
RDT: Rapid diagnostic test
CHIA: Chemiluminescence immunoassay
